# A Case of Dibothriocephalosis (*Dibothriocephalus latus*) from Iseo Lake (Northern Italy): An Update on a Persistent Sanitary Issue

**DOI:** 10.3390/pathogens14010100

**Published:** 2025-01-20

**Authors:** Vasco Menconi, Lisa Guardone, Elena Lazzaro, Romina Bottazzo, Valeria Besutti, Patrizia Danesi, Amedeo Manfrin, Andrea Basso, Giuseppe Arcangeli, Luana Cortinovis, Ewa Bilska-Zając, Giorgia Angeloni

**Affiliations:** 1 Istituto Zooprofilattico Sperimentale delle Venezie, Viale dell’Università, 10, 35020 Legnaro (PD), Italy; vmenconi@izsvenezie.it (V.M.); elazzaro@izsvenezie.it (E.L.); rbottazzo@izsvenezie.it (R.B.); pdanesi@izsvenezie.it (P.D.); amanfrin@izsvenezie.it (A.M.); abasso@izsvenezie.it (A.B.); garcangeli@izsvenezie.it (G.A.); lcortinovis@izsvenezie.it (L.C.); gangeloni@izsvenezie.it (G.A.); 2 FishLab, Department of Veterinary Sciences, University of Pisa, Viale Delle Piagge 2, 56124 Pisa, Italy; 3 Azienda Ospedaliera, Università di Padova, UOC di Microbiologia e Virologia, Via Ospedale Civile, 3651, 35121 Padova, Italy; valeria.besutti@aopd.veneto.it; 4 Department of Parasitology and Invasive Diseases, National Veterinary Research Institute, Partyzantow Avenue 57, 24100 Pulawy, Poland; ewa.bilska@piwet.pulawi.pl

**Keywords:** cestoda, fish-borne zoonosis, food safety, European perch

## Abstract

Dibothriocephalosis is a fish-borne parasitic zoonosis that is caused by tapeworms of the *Dibothriocephalus* (syn. *Diphyllobothrium*) genus. This paper describes a human case of dibothriocephalosis associated with the consumption of a presumably infected fish, prepared at a restaurant near Iseo Lake (northern Italy). A month after, the patient found a segment of a worm in her stool. Molecular analysis identified it as *Dibothriocephalus latus* (syn. *Diphyllobothrium latum*). Several studies reported *D. latus* infections in patients from the subalpine regions of Switzerland, France, and Italy, but no cases have been reported from this area in the last 10 years. This report updates the epidemiology of dibothriocephalosis and emphasises the importance of collaboration among healthcare institutions for a prompt diagnosis and the need for food safety education for Food Business Operators and consumers to reduce transmission risks.

## 1. Introduction

**Figure 1:**
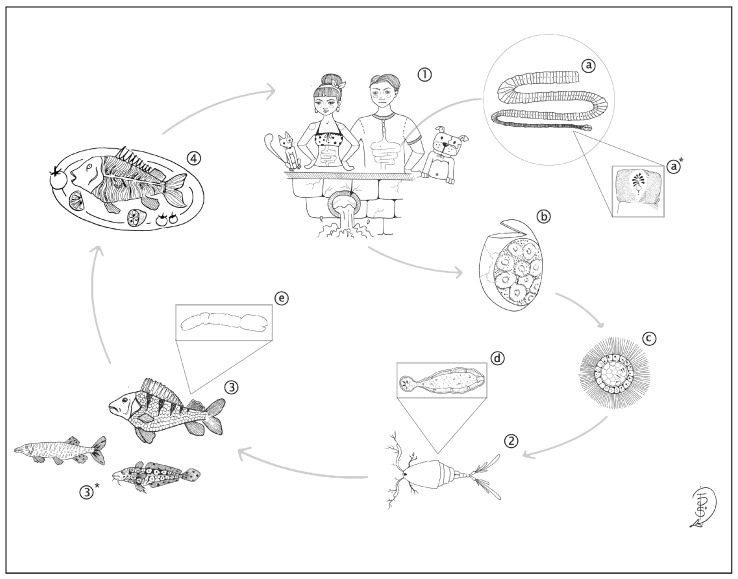
*Dibothriocephalus latus* life cycle: (**1**) mammals, including humans, act as definitive hosts; (**2**) crustacean copepods act as I intermediate hosts; (**3**) the European perch act as II intermediate host; (3*) burbot and pike act as paratenic hosts; (**4**) dish including undercooked perch fillet; (**a**) adult worm; (a*) details of a proglottid; (**b**) egg; (**c**) coracidia; (**d**) procercoid larvae; (**e**) plerocercoid larvae.

The global fisheries and aquaculture production provide a valuable source of animal protein, but fish consumption can pose a risk of infections from bacterial, viral, and parasitic species. Fish-borne parasitic helminths are transmitted by consuming raw or undercooked fish dishes (such as ceviche, sushi, and sashimi) that are contaminated with vital parasite larvae. The growing interest in dishes from different regions of the world has been described as one of the key factors of the increased presence and wider geographical distribution of zoonotic fish parasites [1]. Dibothriocephalosis (formerly known as diphyllobothriosis or, incorrectly, diphyllobothriasis) is caused by cestodes of the order Diphyllobothriidea and affects approximately 20 million people worldwide. *Dibothriocephalus latus* (syn. *Diphyllobothrium latum*) is the primary agent of human dibothriocephalosis [2]. The life cycle of *D. latus* is indirect, with two aquatic intermediate hosts (crustacean copepods and freshwater fish) and fish-eating mammals, humans included, acting as the final host (Figure 1). Moreover, recent studies suggest that wild or domesticated carnivores may play a minor role in maintaining the parasite’s cycle [2]. Eggs pass into the aquatic environment through the definitive host’s faeces, hatch, and release the coracidium. The coracidium attracts copepod (I intermediate host) predation by swimming, and once ingested, it develops into a procercoid larva. When a fish preys on the infected copepod, the procercoid migrates towards the internal organs of the fish, mainly to the muscular tissue, and evolves into the infective stage of plerocercoid [3]. In Europe, the European perch (*Perca fluviatilis*) acts as the main second intermediate host, while pike (*Esox lucius*) and burbot (*Lota lota*) or larger perch may enter the cycle as paratenic hosts [4]. In the fillets, larvae are easily detectable with the naked eye and appear whitish, similar to a boiled grain of rice (0.5–1 cm). Several studies have reported cases of dibothriocephalosis in the Alpine lake region of Switzerland, France, and Italy over the past century and until 2015 [5,6,7,8]. Indeed, this study aims to report and describe a human case of *D. latus* infection from an Alpine lake (Iseo lake), updating the epidemiology of the parasite and demonstrating that it is a persistent public health issue in the region.

## 2. Case Report

**Figure 2:**
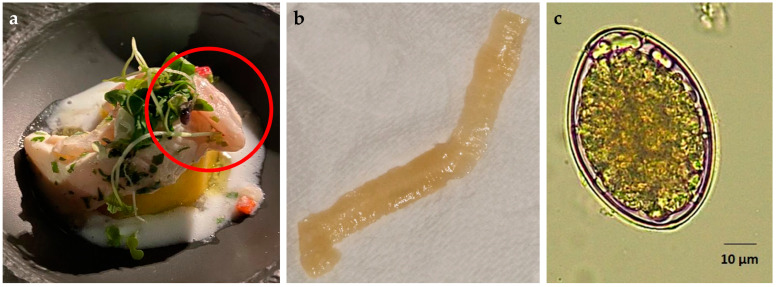
(**a**) Dish consisting of a composition of perch fillet, photographed and consumed by the patient. In the red circle, the fish meat clearly appears raw. (**b**) The tapeworm segment (fragment of the proglottid chain, 12–15 cm) retrieved by the patient from the stools. (**c**). *Dibothriocephalus latus* egg found by sedimentation of a sample of the patient’s stool before the treatment.

On 20 September 2023, a 49-year-old woman in overall good health found a white tape-like worm segment, approximately 12–15 cm in length, in her stool (Figure 2). The patient took the finding to the Parasitology Laboratory of the Istituto Zooprofilattico Sperimentale delle Venezie (IZSVe), where she was advised to consult a physician for further evaluation. The physical examination showed no significant findings, and the patient did not exhibit any symptoms. During the anamnestic interview, she stated that she had consumed a typical European perch dish (Figure 2) at a restaurant near Iseo Lake (Lombardy, Italy) on August 19th. The patient did not report any travel abroad in the last four years, nor the habitual consumption of fish products. The practitioner prescribed her coprological examination and blood tests, which were performed on 7 October 2023 at the Microbiological Unit of Padua University Hospital. The blood tests showed an elevated level of eosinophilic leucocytes (1.21 × 10^9^/L; normal range: 0–0.50 × 10^9^/L), whereas all other parameters included in the Complete Blood Count fell within normal ranges. It is impossible to infer information about any weight loss from the patient, because she had been on a slimming diet for months. A parasitological analysis of faecal samples was performed using the standard sedimentation technique, which revealed the presence of parasite eggs. The retrieved worm segment was examined at IZSVe using a stereomicroscope, revealing a worm strobila without a scolex. The gravid proglottids were wider than they were long, displaying a rosette-shaped structure (uterus). Under the light microscope, eggs discharged by proglottid were oval and brown, with an operculum at the narrowed pole and average size of 68 × 49 µm (n = 30; range: 63–73 × 47–51 µm) (Figure 2). Based on Scholtz et al. [9], the eggs and proglottids were morphologically identified as belonging to the Diphyllobothriidae family. The proglottids were preserved in absolute ethanol at 4 °C for molecular analysis. Total DNA was extracted from two of them using the QIAamp DNA Mini Kit (Qiagen GmbH, Hilden, Germany), following the manufacturer’s protocol for animal blood and tissues. The concentration and purity of the extracted DNA were assessed using a NanoDrop Lite Spectrophotometer (Thermo Fisher Scientific Inc., Waltham, MA, USA). End-point PCR targeting the *cox*1 mitochondrial gene was performed on a Bio-Rad CFX96 Thermal Cycler (Bio-Rad Laboratories, Hercules, CA, USA) using specific primers for cestodes, as described by Bowles et al. [10]. Each 25 µL reaction contained 1× Buffer, 2 mM MgCl_2_, 0.8 mM dNTPs, 0.2 µM of each primer, and 2 U of Platinum™ Taq DNA Polymerase (Thermo Fisher Scientific). For each sample, 2 µL of template DNA diluted at 50 ng/µL was added to the mixture. The thermal cycling protocol consisted of an initial denaturation at 94 °C for 2 min, followed by 35 cycles of denaturation at 94 °C for 30 s, annealing at 50 °C for 30 s, extension at 72 °C for 30 s, and a final extension step at 72 °C for 5 min. PCR products were visualised on a 1% agarose gel (Sigma-Aldrich, St Louis, MO, USA) that was stained with GelRed (Biotium, San Francisco, CA, USA). Amplicons of the expected length (approximately 350–400 nt) were sequenced in-house using an ABI PRISM 3500xl Genetic Analyzer (Applied Biosystems, Foster City, CA, USA) with the BigDye Terminator v3.1 Cycle Sequencing Kit. The obtained sequences, trimmed of primers, were identical and were used to generate a consensus sequence of 396 nt, which was compared by BLASTn analysis in the GenBank database. The obtained consensus sequence (deposited in GenBank under the accession number PQ827203) matched the *cox*1 of *Dibothriocephalus latus* with 100% similarity (best match: OR766558—*D. latus* found in an owned labrador retriever from Italy). At the same time, the analysis showed nucleotide differences above 6% compared to other congeneric species (e.g., *Dibothriocephalus dendriticus*, MW979714), indicating a clear gap that was useful for identifying *D. latus* by sequence comparison. On 3 November 2023, the patient received a single oral dose of praziquantel (600 mg), and on 7 December 2023, the coprological examination was negative for the presence of *D. latus* eggs and proglottids. The patient consented to the publication of this report.

## 3. Discussion

The patient showed no symptoms, in agreement with literature data, which indicates that dibothriocephalosis is typically asymptomatic. This characteristic can contribute to the underdiagnosis and underestimation of human cases [11]. When present, the most reported symptoms include nausea, diarrhoea, abdominal discomfort, and weight loss [9]. Rarely, severe infections can cause intestinal obstructions and cholangitis or cholecystitis due to proglottids migrating to the cholecystic duct [12]. Moreover, even if rarely, *D. latus* is known to interfere with the vitamin B12–intrinsic factor complex within the intestine, hindering the host’s ability to absorb vitamin B12. Prolonged infections may result in megaloblastic anaemia, even if this complication has only been sporadically reported since the 1980s [9]. Despite being rare, vitamin B12 deficiency in mothers can cause risky complications during pregnancy for both mother and foetus, with maternal peripheral neuropathy, while the foetus can suffer anomalies in the development of the nervous system and a reduction in intra-uterine growth [13]. Praziquantel is administered to treat dibothriocephalosis, and a single oral dose of 25 mg/kg has proven to be highly effective and safe, also during pregnancy and lactation [9,13]. The only alteration shown by the patient was an elevated level of eosinophilic leucocyte. Eosinophilia is a marker of parasitic infections; however, it is neither sensitive, nor specific, for a particular parasite species. A significantly elevated eosinophil count may reflect a high parasite load, but the correlation is unreliable [14]. The diagnosis of dibothriocephalosis relies on finding eggs or chains of proglottids in stool specimens; therefore, the coprological examination is widely used as a non-invasive diagnostic tool. Occasionally, *D. latus* infections have been diagnosed during colonoscopy [11]. Hospital laboratories generally identify dibothriocephalid worms according to the morphological features of eggs and worm segments. However, morphological criteria can be insufficient for genus differentiation and specific identification. Species-level identification of parasites by molecular techniques has proven critical for epidemiological studies, discriminating autochthonous infections from non-native ones. The *cox*1 gene has been established as a reliable diagnostic marker, leading to the development of several multiplex PCR methods for the simultaneous and differential identification of diphyllobothriid species [7]. As mentioned, dibothriocephalosis is recognised as a public health problem, especially in the subalpine region [2]. The most recent cases are six human cases diagnosed in France (Haute-Savoie) during an epidemiological survey that was conducted between 2011 and 2013 [8]. Additionally, recent data from Italy were collected through a survey funded by the Italian Ministry of Health, which recorded ten human cases in the subalpine lake region between 2012 and 2015 [Vasco Menconi personal communication]. Unfortunately, such surveys, as well as hospital reports, are often not published, and the available data are fragmentary and show fluctuations over the decades. Notably, the lack of information on human cases does not necessarily indicate the absence of the parasite in an area. Several studies describe the presence of *D. latus* in the fish population of subalpine lakes [1]. For example, the most recent data on the presence of *D. latus* in *P. fluviatilis* from Lake Iseo range from 6% in the central area to 20% in the northern area [3]. The persistence of *D. latus* is related to several factors, such as the extremely high reproductive potential of *D. latus* (approx. 1 million eggs per day) and the faecal contamination of water bodies. Moreover, the growing trend of consuming raw or undercooked dishes is crucial in parasite transmission [4]. The plerocercoid larvae in fish products are whitish and easily detectable with the naked eye; thus, accurate visual inspection of meat that is destined for risky preparation is recommended. The parasite can be inactivated by freezing at −18 °C for 24 h and by cooking to a core temperature above 56 °C [15]. The presence of visible parasites may alter the commercial quality of fishery products [16], making them unfit for the market. According to European legislation, Food Business Operators (FBOs) must ensure that fishery products have been subjected to a visual examination to detect visible parasites (“with dimension, colour or texture which is clearly distinguishable from fish tissues”) before being placed on the market. Fishery products that are contaminated with parasites should not be placed on the market for human consumption [17,18]. Moreover, fish products that are intended to be eaten raw or not fully cooked should be submitted to a cold treatment [19]. The great subalpine lakes are renowned touristic locations and attract tourists for traditional cuisine and recreational fishing, which could facilitate and contribute to the spread of human dibothriocephalosis. Although *D. latus* is the primary agent of dibothriocephalosis in Europe, recent studies reported cases of human infection in France, Switzerland, and Spain that were caused by non-native species such as *D. nihonkaiense*, *D. dendriticum,* and *D. pacificum* [20,21,22,23,24,25]. These cases have been linked either to the consumption of imported fish products or to the international mobility of people from endemic areas, highlighting the risk of introducing and spreading new parasite species due to the global fish trade and human travelling and migration [1,20,21,22,23,24,25]. This report emphasises the importance of collaboration between experts and health institutions in managing zoonotic diseases. A multidisciplinary approach, incorporating clinical evaluation, epidemiological data, and molecular techniques, ensures an accurate diagnosis that leads to an effective treatment. Additionally, the anamnestic investigation, focusing on the patient’s travel history and dietary habits, is essential to identify the source of infection. To reduce the risk of transmission, it is essential to correctly apply food safety regulations and prevent faecal contamination of reservoirs, with special attention being paid to locations where human cases and a high prevalence of host fish have been recorded.

## Data Availability

The original contributions presented in this study are included in the article. Further inquiries can be directed to the corresponding author.
